# Green and Blue Spaces and Behavioral Development in Barcelona Schoolchildren: The BREATHE Project

**DOI:** 10.1289/ehp.1408215

**Published:** 2014-09-09

**Authors:** Elmira Amoly, Payam Dadvand, Joan Forns, Mónica López-Vicente, Xavier Basagaña, Jordi Julvez, Mar Alvarez-Pedrerol, Mark J. Nieuwenhuijsen, Jordi Sunyer

**Affiliations:** 1 Centre for Research in Environmental Epidemiology (CREAL), Barcelona, Spain; 2 Universitat Pompeu Fabra (UPF), Barcelona, Spain; 3 Universidad Autónoma de Barcelona, Cerdanyola del Vallès, Spain; 4 CIBER Epidemiología y Salud Pública (CIBERESP), Barcelona, Spain; 5 Department of Genes and Environment, Division of Epidemiology, Norwegian Institute of Public Health, Oslo, Norway; 6 IMIM (Hospital del Mar Medical Research Institute), Barcelona, Spain

## Abstract

Background: Green spaces have been associated with improved mental health in children; however, available epidemiological evidence on their impact on child behavioral development is scarce.

Objectives: We investigated the impact of contact with green spaces and blue spaces (beaches) on indicators of behavioral development and symptoms of attention deficit/hyperactivity disorder (ADHD) in schoolchildren.

Methods: This study was based on a sample of 2,111 schoolchildren (7–10 years of age) from 36 schools in Barcelona in 2012. We obtained data on time spent in green spaces and beaches and Strengths and Difficulties Questionnaires (SDQ) from parents, and ADHD/DSM-IV questionnaires from teachers. Surrounding greenness was abstracted as the average Normalized Difference Vegetation Index (NDVI) in buffers of 100 m, 250 m, and 500 m around each home address. Proximity to green spaces was defined as living within 300 m of a major green space (≥ 0.05 km^2^). We applied quasi-Poisson mixed-effects models (with school random effect) to separately estimate associations between indicators of contact with green spaces and SDQ and ADHD total and subscale scores.

Results: We generally estimated beneficial associations between behavioral indicators and longer time spent in green spaces and beaches, and with residential surrounding greenness. Specifically, we found statistically significant inverse associations between green space playing time and SDQ total difficulties, emotional symptoms, and peer relationship problems; between residential surrounding greenness and SDQ total difficulties and hyperactivity/inattention and ADHD/DSM-IV total and inattention scores; and between annual beach attendance and SDQ total difficulties, peer relationship problems, and prosocial behavior. For proximity to major green spaces, the results were not conclusive.

Conclusion: Our findings support beneficial impacts of contact with green and blue spaces on behavioral development in schoolchildren.

Citation: Amoly E, Dadvand P, Forns J, López-Vicente M, Basagaña X, Julvez J, Alvarez-Pedrerol M, Nieuwenhuijsen MJ, Sunyer J. 2014. Green and blue spaces and behavioral development in Barcelona schoolchildren: the BREATHE Project. Environ Health Perspect 122:1351–1358; http://dx.doi.org/10.1289/ehp.1408215

## Background

Contact with green spaces has been reported to improve both perceived and objective physical and mental health and well-being (Bowler et al. 2010). Underlying mechanisms of health benefits of green spaces are not well understood, but research suggests that increasing physical activity, restoring psychological stress, reducing anxiety and depression, increasing social contacts/cohesion, reducing noise and air pollution levels, and moderating ambient temperature may underlie such benefits (Bowler et al. 2010; Dadvand et al. 2012b; Lee and Maheswaran 2011; Maas et al. 2009a). Although contact with green spaces could also influence behavioral development through some of these pathways (e.g., increasing physical activity and social contacts and reducing stress and exposure to noise and air pollution) (Freire et al. 2010; Goodman et al. 2007; Huby et al. 2006; Tiesler et al. 2013; Ussher et al. 2007; van Kempen et al. 2012; Wachs et al. 2009; Watkins et al. 2013), the available epidemiological evidence on such an impact is scarce.

Neuropsychiatric problems including behavioral problems occur in 10–20% of children worldwide (Kieling et al. 2011). The most commonly diagnosed behavioral disorder among children and adolescents is attention deficit/hyperactivity disorder (ADHD), which has an estimated global prevalence of 5.3% (Polanczyk et al. 2007). In Spain ADHD has been reported to affect 6.8% of children and adolescents (Catalá-López et al. 2012). Behavioral problems are accompanied by a considerable burden for individuals, families, and society because such problems are associated with both short-term (e.g., impaired school attainment) and long-term (e.g., mental health problems in adulthood) consequences (Kieling et al. 2011).

In this study we aimed to investigate the association, if any, between contact with green and blue (beaches) spaces and indicators of behavioral development and symptoms of ADHD in schoolchildren.

## Materials and Methods

*Study population.* This cross-sectional study was conducted as part of the BRain dEvelopment and Air polluTion ultrafine particles in scHool childrEn (BREATHE) project. The data collection for our study was carried out between January 2012 and March 2013. Of the 416 schools in the city of Barcelona, 36 were selected in order to obtain maximum contrast in air pollution levels [i.e., nitrogen dioxide (NO_2_)] at schools. Additionally, three more schools from Sant Cugat del Vallès, a town located 20 km northwest of Barcelona, were also included in BREATHE (39 schools in total). Participating schools were similar to the remaining schools in Barcelona in terms of the socioeconomic vulnerability index (0.66 vs. 0.62, *p* = 0.20) and NO_2_ levels (51.5 vs. 50.9 μg/m^3^, *p* = 0.81). Families of schoolchildren in the second to fourth grades (7–10 years of age) of these schools were invited to participate in the study by letters and/or presentations in schools, and 2,897 (59%) agreed to take part in BREATHE. Data from one school suggested that participating children had a higher good or excellent school achievement rate (53.5% vs. 39.3%) and a lower prevalence of ADHD (8.1% vs. 11.4%) compared with nonparticipating children. Because of unavailability of a detailed green space map for Sant Cugat del Vallès, we limited this current analysis to the BREATHE participants from Barcelona schools and excluded 274 participants from schools in Sant Cugat del Vallès.

*Questionnaires.* The parents were asked to fill out BREATHE questionnaires on sociodemographic and household characteristics and the time spent playing in green and blue spaces. We used two sets of questionnaires for behavioral assessment of children: Strengths and Difficulties Questionnaires (SDQ) (Goodman 1997) rated by parents, and ADHD symptom criteria of *Diagnostic and Statistical Manual of Mental Disorders, Fourth Edition* (ADHD/DSM-IV) rated by teachers (American Psychiatric Association 2000). The SDQ questionnaire consists of four difficulties subscales including emotional symptoms, conduct problems, hyperactivity/inattention, and peer relationship problems, and a strengths subscale for prosocial behavior. Each subscale comprises five items that can be scored 0, 1, or 2, and each subscale score can therefore range from 0 to 10. An SDQ total difficulties score (range, 0–40) was calculated by summing the scores of the four difficulties subscales (i.e., all subscales but prosocial) (Goodman 1997). Higher scores for total difficulties and individual difficulties subscales indicate more behavioral problems. On the other hand, higher scores for prosocial behavior indicate better behavioral development.

The ADHD/DSM-IV symptoms consist of inattention and hyperactivity-impulsivity symptoms, each having nine items (18 items in total) (American Psychiatric Association 2000). Each item was scored between 0 and 3 (from no symptoms to severe symptoms), so the scores for ADHD symptoms could range between 0 and 54, and scores for inattention and hyperactivity-impulsivity symptoms could range from 0 to 27. Higher scores for ADHD, inattention, and hyperactivity-impulsivity indicate more behavioral problems.

*Exposure assessment.* Our exposure assessment was carried out as a part of the Positive Health Effects of the Natural Outdoor Environment in Typical Populations of Different Regions in Europe (PHENOTYPE) study (Nieuwenhuijsen et al. 2014) and encompassed three aspects of contact with green spaces: *a*) the time spent playing in green spaces, *b*) residential surrounding greenness, and *c*) residential proximity to a major green space. For the assessment of exposure to blue spaces, we focused on time spent at beaches and residential proximity to beaches.

Green space playing time. Parents were asked to report the average number of times per week and the average number of hours spent each time their child played in green spaces during four different time periods: school days, weekends, and New Year/Christmas and Easter holidays during the last school period, and during the previous summer holiday (see Supplemental Material, “Green Spaces Questionnaire”). For each time period, the total duration of time spent playing in green spaces was calculated by first multiplying the number of play times per week by the hours per play time to obtain weekly average time spent playing in green spaces, and then multiplying these weekly average times by the number of corresponding weeks in that category. Green space playing time was then defined as the annual total time (hours) of playing in green spaces estimated by summing up total duration of playing at green spaces during school days, weekends, New Year/Christmas and Easter holidays, and summer holidays.

**Figure 1:**
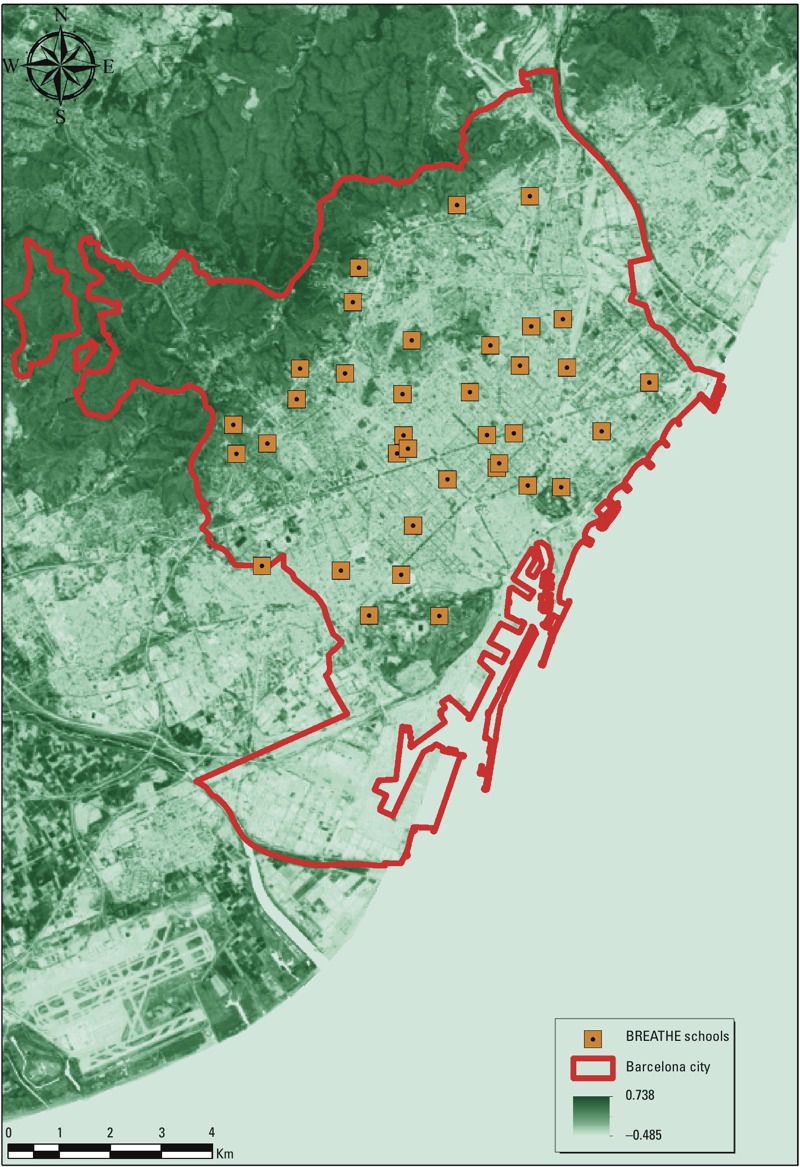
NDVI map of Barcelona, 27 April 2011. Source: Landsat 5 TM data, NASA (https://earthdata.nasa.gov/)

Residential surrounding greenness. Our assessment of residential surrounding greenness was based on the Normalized Difference Vegetation Index (NDVI) derived from the Landsat 5 Thematic Mapper (TM) data at 30 m × 30 m resolution (Weier and Herring 2011). NDVI is an indicator of greenness based on land surface reflectance of visible (red) and near-infrared parts of spectrum (Weier and Herring 2011). It ranges between –1 and 1, with higher numbers indicating more greenness. To achieve maximum exposure contrast, we looked for available cloud-free Landsat TM images during springs/autumns (i.e., the maximum vegetation period of the year for our study region) of 2011–2012 (the relevant years to our study period) from the NASA’s Earth Observing System Data and Information System (EOSDIS) website (https://earthdata.nasa.gov/). On the basis of this search we generated our NDVI map using the image obtained on 27 April 2011 (Figure 1).

Surrounding greenness was abstracted as the average of NDVI in buffers of 100 m, 250 m, and 500 m (Dadvand et al. 2012a, 2012b, 2012c; Lovasi et al. 2011; Markevych et al. 2014) around the main home address of each study participant, which was geocoded according to the address when the questionnaires were completed. We used different buffer sizes to abstract residential surrounding greenness in order to explore the consistency of our findings across different buffer sizes. Furthermore, we hypothesized that immediate surrounding greenness (e.g., 100-m buffer) could be more relevant to mechanisms such as psychological restoration (because of visual access to greenness) and reduction in environmental exposure (e.g., air pollution and noise), whereas greenness in larger buffer sizes might be more likely to be associated with other mechanisms, such as increased physical activity.

**Figure 2:**
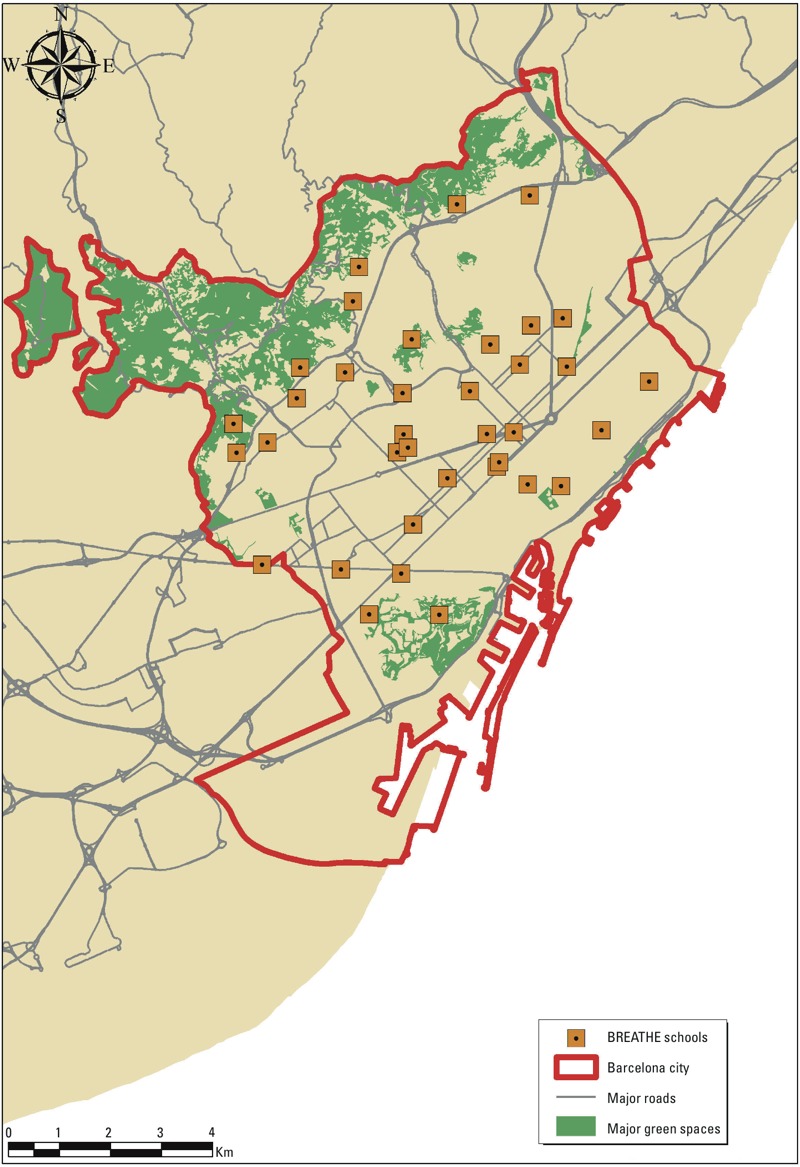
Major green spaces across Barcelona. Source: Ecologic Map of Barcelona (Burriel 2004).

Residential proximity to major green spaces. We hypothesized that proximity to green spaces could act as a surrogate for access to green spaces (European Commission, Expert Group on the Urban Environment 2001). To define the green spaces we used the Ecological Map of Barcelona (Burriel 2004; Dadvand et al. 2012a). From this map we constructed a binary variable (yes/no) indicating whether the child’s home address was within 300 m of a major green space, defined as a green space with an area ≥ 0.05 km^2^, which roughly corresponds to the 75th percentile of the area of green spaces across our study region (Dadvand et al. 2012a) (Figure 2). The 300-m distance was selected according to the European Commission recommendation for access to green spaces (European Commission, Expert Group on the Urban Environment 2001).

*Main analyses.* We conducted complete case analyses without imputing missing data. For each exposure–outcome pair, we developed quasi-Poisson mixed-effects models, with school as random effect, SDQ or ADHD scores as outcomes, and green space playing time, residential surrounding greenness, or proximity to a major green space (one at a time) as fixed-effect predictors. We separately assessed scores of SDQ total difficulties and its subscales (emotional symptoms, conduct problems, hyperactivity/inattention, peer relationship problems, and prosocial behavior) and scores for ADHD, inattention, and hyperactivity-impulsivity symptoms as the outcomes. Analyses were adjusted for the following *a priori* covariates: child’s sex (male/female), school level (second/third/fourth grade), ethnicity (Spanish/non-Spanish), preterm birth (yes/no), breastfeeding (6 months/6–12 months/no breastfeeding), exposure to environmental tobacco smoke (yes/no), maternal smoking during pregnancy (yes/no), responding person (mother/father/other), parental educational achievement (none or primary education/secondary education/university), parental employment status (unemployed/employee/self-employed), parental marital status (single/non-single), and neighborhood socioeconomic status based on quartiles of 2010 household income (renta familiar disponible) (Generalitat de Catalunya 2010). For green space playing time and residential surrounding greenness, we have reported the percent change in average outcome scores associated with a 1–interquartile range (IQR) increase (based on all study participants) in annual total time playing in green spaces and average NDVI, respectively.

*School greenness and combined home–school greenness.* We separately explored the impacts of school greenness and combined home–school greenness. Because we did not have school building plans, we averaged NDVI over a 100-m buffer around the school central point, assuming that it can capture all the school premises. We then abstracted a home–school greenness index by averaging residential surrounding NDVI (100-m buffer) and school greenness weighted by the daily time that children were assumed to spend at home (16 hr) and at school (8 hr). We repeated the main analyses, using school greenness and home–school greenness index (one at a time) as alternative exposures.

*Blue space.* Parents were asked to report how many days (on average) they went with their child to the beach for leisure during the school period (September–June) and summer holidays (June–September). We calculated annual beach attendance (days) by adding these two numbers and repeated the main analyses using this as an alternative exposure.

Of 2,110 participants, 11 (0.5%) and 35 (1.7%) lived within 300 m and 500 m of the beach respectively. Therefore, it was not feasible to use proximity to blue spaces as a predictor in our analyses.

*Ethics approval.* All parents and tutors signed the informed consent and the study was approved (No. 2010/41221/I) by the Clinical Research Ethical Committee of the Parc de Salut MAR, Barcelona, Spain.

## Results

*Study population.* Of 2,897 BREATHE participants, 2,623 were from schools in Barcelona. Of these, 2,111 with available data from all three questionnaires (i.e., SDQ, ADHD/DSM-IV, and BREATHE general questionnaires) and geocoded home addresses were included in the analyses. Sociodemographic characteristics of the BREATHE participants and those included in this current study are presented in Table 1.

**Table 1:** Description of characteristics of all BREATHE participants (*n* = 2,897) and those included in this current study (*n* = 2,111), Barcelona, 2012 [*n* (%)].

Variable	BREATHE participants	Study participants
Child sex
Female	1,441 (49.7)	1,040 (49.3)
Male	1,456 (50.3)	1,071 (50.7)
Child school level (grade)
Second	1,083 (37.4)	808 (38.2)
Third	1,036 (35.8)	750 (35.5)
Fourth	778 (26.9)	553 (26.2)
Maternal educational achievement
None or primary education	347 (12.0)	217 (10.3)
Secondary education	781 (27.0)	618 (29.3)
University education	1,596 (55.1)	1,260 (59.7)
Missing	173 (5.9)	16 (0.8)
Paternal educational achievement
None or primary education	419 (14.5)	262 (12.4)
Secondary education	848 (29.3)	652 (30.9)
University	1,418 (49.0)	1,154 (54.7)
Missing	212 (7.3)	43 (2.0)
Maternal employment status
Unemployed	476 (16.4)	358 (17.0)
Employee	1,781 (61.5)	1,373 (65.0)
Self-employed	453 (15.7)	352 (16.7)
Missing	187 (6.5)	28 (1.3)
Paternal employment status
Unemployed	260 (9.0)	212 (10.0)
Employee	1,565 (54.0)	1,200 (56.9)
Self-employed	834 (28.8)	637 (30.2)
Missing	238 (8.2)	62 (2.9)
Adopted child
Yes	105 (3.6)	84 (4.0)
No	2,616 (90.3)	2,007 (95.1)
Missing	176 (6.1)	20 (1.0)
Parental marital status
Non-single parent	2,312 (79.8)	1,770 (83.9)
Single parent	411 (14.2)	325 (15.4)
Missing	174 (6.0)	16 (0.8)
Exposure to environmental tobacco smoke
Yes	337 (11.6)	275 (13.0)
No	2,382 (82.2)	1,814 (85.9)
Missing	178 (6.1)	22 (1.0)
Maternal smoking during pregnancy
Yes	261 (9.0)	210 (10.0)
No	2,395 (82.7)	1,831 (86.7)
Missing	241 (8.3)	70 (3.3)
Preterm birth
Yes	200 (6.9)	159 (7.5)
No	2,434 (84.0)	1,867 (88.0)
Missing	263 (9.1)	86 (4.1)
Breastfeeding
No	476 (16.4)	394 (18.7)
< 6 months	1,422 (49.1)	1,074 (50.9)
> 6 months	788 (27.2)	597 (28.3)
Missing	211 (7.3)	46 (2.2)
Ethnicity
Spanish	2,574 (88.9)	1,908 (90.4)
Non-Spanish	279 (9.6)	189 (9.0)
Missing	44 (1.5)	14 (0.7)

*Questionnaires.* The median (IQR) for SDQ total difficulties score was 8 (7). For SDQ subscales the median (IQR) scores for prosocial behavior, hyperactivity/inattention, emotional symptoms, conduct problems, and peer relationship problems were respectively 9 (2), 3 (3), 2 (2), 1 (3), and 1 (2). The median (IQR) scores for ADHD, inattention, and hyperactivity-impulsivity symptoms were 5 (11), 3 (8), and 1 (3), respectively.

Cronbach’s alpha coefficient (an indicator of internal consistency) for SDQ total difficulties was 0.80, and for subscales of prosocial behavior, hyperactivity/inattention, emotional symptoms, conduct problems, and peer relationship problems was 0.62, 0.77, 0.59, 0.57, and 0.55, respectively. The Cronbach’s alpha coefficient for scores of ADHD, inattention, and hyperactivity-impulsivity symptoms was 0.95, 0.94, and 0.93, respectively. The Spearman correlation coefficient between SDQ hyperactivity/inattention subscale score (rated by parents) and ADHD symptoms score (rated by teachers) was 0.44.

*Exposure assessment.* The median (IQR) of green space playing time was 480 (492) hr/year. Regarding surrounding greenness, the median (IQR) of the average NDVI across buffers of 100 m, 250 m, and 500 m around home addresses was 0.044 (0.058), 0.051 (0.047), and 0.055 (0.049), respectively. Of 2,111 study participants included in analyses, 384 (18.1%) were living within 300 m of a major green space. Those living within 300 m of a major green space had higher residential surrounding greenness compared with those living further away (see Supplemental Material, Table S1).

On average, an IQR increase in residential surrounding greenness over buffers of 100 m, 250 m, and 500 m was associated, respectively, with use of green spaces during 28.6 [95% confidence interval (CI): 13.1, 44.2], 26.7 (95% CI: 13.6, 39.9), and 31.9 (95% CI: 18.8, 45.1) more hours per year. Living within 300 m of a major green space was associated with a statistically nonsignificant 18.3 hr/year (95% CI: –19.6, 56.3) increase in using green space compared to those living further away.

In general, children of parents with lower educational achievement, unemployed parents, single parents, and non-Spanish origin had less green space playing time and higher scores for SDQ total difficulties and ADHD symptoms (i.e., more behavioral problems) (see Supplemental Material, Table S2). However, there was not any notable difference in residential surrounding greenness across strata of parental educational achievements, employment status, marital status, and ethnicity.

*Main analyses.* Green space playing time. We estimated inverse associations between green space playing time and scores of SDQ total difficulties and difficulties subscales (except for conduct problems) and a weak positive association between this exposure and the strengths subscale score (Table 2). Specifically, we observed statistically significant associations between the green space playing time and scores of SDQ total difficulties, emotional symptoms and peer relationship problems (Table 2).

**Table 2:** Unadjusted and adjusted*^a^* percent change (95% CI) in outcomes associated with an IQR increase in the green space playing time (492 hr) and annual beach attendance (32 days) and with living within 300 m of a major green space (residential proximity), Barcelona, 2011.

Outcome	Green space playing time	Annual beach attendance	Residential proximity
SDQ
Difficulties
Total
Unadjusted	–6.6 (–10.1, –2.9)**	–4.3 (–7.5, –0.9)**	–0.3 (–7.3, 7.2)
Adjusted	–4.8 (–8.6, –0.9)**	–3.9 (–7.2, –0.4)**	–1.3 (–8.2, 6.2)
Hyperactivity/inattention
Unadjusted	–4.0 (–8.1, 0.2)*	–0.6 (–4.3, 3.2)	–1.8 (–9.4, 6.3)
Adjusted	–2.7 (–7.0, 1.5)	–0.7 (–4.6, 3.2)	–3.0 (–10.6, 5.2)
Emotional symptoms
Unadjusted	–11.6 (–16.6, –6.2)**	–6.6 (–11.4, –1.6)**	4.8 (–5.6, 16.4)
Adjusted	–8.2 (–13.9, –2.2)**	–3.9 (–9.1, 1.6)	1.9 (–8.7, 13.8)
Conduct problems
Unadjusted	–3.7 (–9.7, 2.9)	–5.0 (–10.4, 0.7)*	2.3 (–9.2, 15.3)
Adjusted	0.7 (–5.6, 7.5)	–2.8 (–8.2, 2.9)	0.6 (–10.6, 13.1)
Peer relationship problems
Unadjusted	–17.5 (–24.0, –10.4)**	–17.6 (–23.7, –11.1)**	–2.4 (–16.4, 13.8)
Adjusted	–15.4 (–22.7, –7.4)**	–16.8 (–23.4, –9.7)**	–5.1 (–19.1, 11.3)
Strengths
Prosocial behavior
Unadjusted	0.3 (–0.9, 1.5)	1.0 (0.0, 2.1)**	0.9 (–1.3, 3.1)
Adjusted	0.2 (–1.0, 1.5)	1.1 (0.0, 2.2)**	0.7 (–1.5, 3.0)
ADHD/DSM-IV
ADHD
Unadjusted	–1.6 (–8.8, 6.1)	–1.1 (–7.5, 5.8)	4.6 (–8.9, 20.2)
Adjusted	–1.6 (–9.0, 6.4)	–0.1 (–6.7, 6.9)	1.8 (–11.6, 17.3)
Inattention
Unadjusted	–0.7 (–7.8, 7.0)	–3.6 (–9.8, 3.0)	2.6 (–10.6, 17.7)
Adjusted	–0.2 (–7.7, 7.9)	–2.4 (–8.9, 4.5)	–0.2 (–14.3, 23.5)
Hyperactivity-impulsivity
Unadjusted	–4.0 (–13.0, 5.9)	3.2 (–5.2, 12.5)	6.6 (–10.8, 27.3)
Adjusted	–5.4 (–14.5, 4.8)	3.5 (–5.2, 12.9)	2.9 (–14.3, 23.5)
^***a***^Adjusted for child’s school level, sex, ethnicity, preterm birth, breastfeeding, exposure to environmental tobacco smoke, maternal smoking during pregnancy, responding person, parental educational achievement, parental employment status, parental marital status, and neighborhood socioeconomic status. *p < 0.1. **p < 0.05.			

For ADHD, inattention, and hyperactivity-impulsivity symptoms, although higher green space playing time was generally associated with lower scores, the associations did not attain statistical significance (Table 2).

Including an indicator of physical activity (the frequency of physical exercise that could make the child sweating or breathless) in fully adjusted models weakened most of our observed associations for green space playing time (see Supplemental Material, Table S3).

Residential surrounding greenness. Regarding the SDQ and its subscales, we observed consistent patterns of associations across different buffer sizes (Table 3). Higher residential surrounding greenness was associated with lower scores for the SDQ total difficulties and difficulties subscales, and a higher average score for the strengths subscale. We observed statistically significant associations for SDQ total difficulties and hyperactivity/inattention scores for residential surrounding greenness in all buffer sizes, and for emotional symptoms only for residential surrounding greenness in a 500-m buffer (Table 3). Furthermore, there were marginally statistically significant (0.05 < *p* < 0.10) reductions in scores of SDQ conduct problems associated with average NDVI across buffers of 100 m and 250 m (Table 3). Additionally, we estimated lower average scores for peer relationship problems associated with residential surrounding greenness, which were marginally statistically significant for 250-m buffer and statistically nonsignificant for 100-m and 500-m buffers (Table 3).

**Table 3:** Unadjusted and adjusted*^a^* percent change (95% CI) in outcomes associated with an IQR increase in the average of NDVI surrounding participants’ residences, school (school greenness), and home and school combined (home–school greenness), Barcelona, 2011.

Outcome	Residential surrounding greenness			School greenness	Home–school greenness
	100-m buffer	250-m buffer	500-m buffer		
SDQ
Difficulties
Total
Unadjusted	–2.7 (–5.7, 0.3)*	–3.5 (–6.1, –0.8)**	–3.7 (–6.5, –0.9)**	–5.7 (–12.2, 1.3)	–4.4 (–8.3, –0.3)**
Adjusted	–3.6 (–6.6, –0.6)**	–3.8 (–6.4, –1.2)**	–4.0 (–6.7, –1.2)**	–2.3 (–7.4, 3.1)	–4.5 (–8.6, –0.1)**
Hyperactivity/inattention
Unadjusted	–4.4 (–7.6, –1.0)**	–4.2 (–7.1, –1.3)**	–3.9 (–6.9, –0.9)**	–5.0 (–10.7, 1.0)*	–5.8 (–9.7, –1.7)**
Adjusted	–5.0 (–8.2, –1.6)**	–4.5 (–7.4, –1.6)**	–4.1 (–7.1, –1.0)**	–2.1 (–7.3, 3.3)	–5.1 (–9.5, –0.5)**
Emotional symptoms
Unadjusted	–0.1 (–4.5, 4.4)	–1.6 (–5.4, 2.2)	–3.4 (–7.2, 0.6)*	–4.5 (–11.7, 3.3)	–1.5 (–6.8, 4.0)*
Adjusted	–1.4 (–5.9, 3.2)	–2.4 (–6.3, 1.6)	–4.3 (–8.1, –0.1)**	–2.4 (–9.0, 4.6)	–2.5 (–8.3, 3.6)
Conduct problems
Unadjusted	–4.8 (–9.6, 0.3)*	–3.9 (–8.1, 0.5)*	–3.7 (–8.0, 0.9)	–8.1 (–15.9, 0.3)*	–7.2 (–12.9, –1.1)
Adjusted	–4.8 (–9.4, 0.2)*	–3.6 (–7.8, 0.7)*	–3.1 (–7.4, 1.4)	–1.0 (–8.4, 7.0)	–4.3 (–10.6, 2.5)
Peer relationship problems
Unadjusted	–0.4 (–6.7, 6.2)	–3.6 (–9.1, 2.3)	–2.9 (–8.6, 3.3)	–9.8 (–23.2, 5.9)	–2.5 (–10.7, 6.6)
Adjusted	–2.4 (–8.7, 4.3)	–4.9 (–10.4, 0.9)*	–4.6 (–10.2, 1.4)	–4.4 (–13.6, 5.8)	–4.4 (–12.5, 4.4)
Strengths
Prosocial behavior
Unadjusted	0.5 (–0.4, 1.4)	0.7 (–0.1, 1.5)*	0.7 (0.0, 1.5)*	1.1 (–0.3, 2.5)	0.8 (–0.2, 1.9)
Adjusted	0.3 (–0.7, 1.2)	0.5 (–0.3, 1.3)	0.5 (–0.3, 1.3)	0.9 (–0.5, 2.3)	0.7 (–0.5, 2.0)
ADHD/DSM-IV
ADHD
Unadjusted	–2.0 (–7.6, 4.0)	–0.1 (–5.0, 5.0)	0.2 (–4.7, 5.4)	–7.0 (–15.4, 2.3)	–4.2 (–10.7, 2.9)
Adjusted	–6.0 (–11.3, –0.2)**	–2.8 (–7.6, 2.3)	–2.6 (–7.6, 2.7)	–5.4 (–13.6, 3.5)	–7.7 (–14.5, –0.3)**
Inattention
Unadjusted	–2.4 (–7.9, 3.5)	0.3 (–4.5, 5.4)	–0.1 (–5.0, 5.1)	–6.2 (–15.1, 3.8)	–4.2 (–10.9, 2.9)
Adjusted	–6.2 (–11.6, –0.4)**	–1.8 (–6.7, 3.3)	–2.6 (–7.6, 2.7)	–3.9 (–12.5, 5.6)	–7.0 (–14.1, 0.6)*
Hyperactivity-impulsivity
Unadjusted	–1.0 (–8.2, 6.7)	–0.2 (–6.4, 6.4)	1.2 (–5.1, 7.8)	–8.4 (–18.3, 2.8)	–3.9 (–12.1, 5.1)
Adjusted	–5.5 (–12.5, 2.0)	–3.8 (–10.0, 2.8)	–2.3 (–8.8, 4.7)	–7.5 (–17.8, 4.1)	–8.5 (–17.4, 1.3)*
^***a***^Adjusted for child’s school level, sex, ethnicity, preterm birth, breastfeeding, exposure to environmental tobacco smoke, maternal smoking during pregnancy, responding person, parental educational achievement, parental employment status, parental marital status, and neighborhood socioeconomic status. *p < 0.1. **p < 0.05.					

We estimated lower average scores for ADHD, inattention, and hyperactivity-impulsivity symptoms in association with higher surrounding greenness, but the associations were statistically significant only for ADHD and inattention symptoms in relation to residential surrounding greenness in a 100-m buffer (Table 3).

Residential proximity to major green spaces. With regard to SDQ scores, the associations with residential proximity to major green spaces were generally weak, and none attained statistical significance (Table 2). Similarly, residential proximity to major green spaces was not associated with ADHD, inattention, and hyperactivity-impulsivity symptoms (Table 2).

We repeated the analyses using an alternative definition of residential proximity to green spaces as living within 300 m of any green space regardless of its size. Although the associations were generally stronger for this alternative definition compared with those of residential proximity to major green spaces in main analyses, none of the associations attained statistical significance (see Supplemental Material, Table S4).

*School greenness and combined home–school greenness.* The Spearman correlation coefficient of residential surrounding greenness and the school greenness and home–school greenness index was 0.36 and 0.88, respectively. Findings for the school greenness and home–school greenness index were in line with those of main analyses for residential surrounding greenness (Table 3). Home–school greenness index was associated with a statistically significant decrease in scores for SDQ total difficulties and hyperactivity/inattention as well as ADHD symptom scores. We also estimated a nearly significant reduction in average scores for ADHD inattention symptoms and hyperactivity-impulsivity symptoms (Table 3). For the school greenness, although we observed inverse associations with SDQ total difficulties and difficulties subscales as well as ADHD symptoms and a positive association with SDQ strength subscale, none of the associations attained statistical significance.

*Blue space.* The median (IQR) of annual beach attendance was 23 (32) days. The Spearman correlation coefficient between green space playing time and annual beach attendance was 0.19. Annual beach attendance was negatively associated with the SDQ total difficulties score and with difficulty subscale scores (particularly peer relationship problems), and positively associated with the SDQ strength subscale score (i.e., prosocial behavior). However, there was no consistent pattern of associations with ADHD symptom scores.

## Discussion

This is the first epidemiological study to evaluate the association between contact with green and blue spaces and behavioral development in schoolchildren by investigating different aspects of contact with green spaces including use, access, residential surrounding greenness, and school greenness. Some behavioral problem scores were lower in association with the use of green spaces and residential surrounding greenness, but not with residential proximity to major green spaces. We also estimated some beneficial associations between use of blue spaces (i.e., annual days of beach attendance) and behavioral problems.

In our study, SDQ was filled out by parents and ADHD/DSM-IV was reported by teachers. It has been reported that the combination of parents’ and teachers’ reports is more sensitive in detecting behavioral problems than either alone when compared with independent psychiatric assessments (Goodman et al. 2000b). Our analytical strategy led to performing a total of 54 comparisons. Instead of adjusting for multiple comparisons, we based the interpretation of our findings on their consistencies among our different measures of contact with green spaces and our hypothesized mechanisms (Feise 2004; Rothman 1990). Residential surrounding greenness and use of green spaces were inversely associated with SDQ scores for total difficulties and for most difficulties subscales, and with scores for ADHD and inattention symptoms. For these indicators of contact with green spaces, we also estimated nonsignificant associations with SDQ scores for prosocial behavior, a strengths subscale. These findings suggest beneficial impacts (i.e., fewer behavioral problems and more behavioral strengths) associated with these measures of contact with greenness. For SDQ total difficulties and subscales, the associations were generally consistent for residential surrounding greenness across different buffers. For ADHD symptoms and subscales the associations tended to be stronger for immediate residential surrounding greenness (i.e., 100-m buffer). Although green space playing time increased and behavioral problem scores decreased as indicators of socioeconomic status (SES) increased, we did not observe notable differences in residential surrounding greenness according to SES. Therefore, the consistency of our findings for green space playing time and residential surrounding greenness suggests that associations with green space playing time were unlikely to be explained by bias due to residual confounding by SES alone.

We are not aware of any available report on the impacts of contact with green and blue spaces on SDQ scores; therefore, it is not possible to compare our findings with those of others. However, our findings are consistent with several proposed hypotheses. Playing in green spaces has been suggested to contribute to mental well-being in children, including better behavioral, motor, and cognitive development and improved creativity and social skills (Huby et al. 2006). A previous study has reported an inverse association between physical activity and SDQ total difficulties, emotional symptoms, and peer relationship difficulties (Ussher et al. 2007)—the same indicators for which we found an inverse association with green space playing time. Including an indicator of the frequency of physical activity in fully adjusted models for green space playing time weakened most of our observed associations, suggesting that these associations could be mediated partly by physical activity. However, the associations remained statistically significant for SDQ scores for total difficulties, emotional symptoms, and peer relationship problems after further adjustment for physical activity, suggesting that mediators other than physical activity also may have been involved in these associations. Parental and child psychological stress and depression have been reported to be adversely associated with behavioral development (Goodman et al. 2007; Wachs et al. 2009; Watkins et al. 2013), and green spaces have been associated with evidence of stress restorative effects and reduced depression (Bowler et al. 2010; Maas et al. 2009b; Wells and Evans 2003). Furthermore, higher ambient noise has been associated with higher scores for SDQ hyperactivity/inattention and emotional symptoms (Tiesler et al. 2013). The ability of green spaces to reduce noise (De Ridder et al. 2004; Gidlöf-Gunnarsson and Öhrström 2007) might therefore explain a part of our observed reduction in SDQ hyperactivity/inattention and emotional symptoms associated with higher residential surrounding greenness. Similarly, exposure to air pollution has been adversely associated with neurodevelopment (Freire et al. 2010; van Kempen et al. 2012), and residential surrounding greenness has been suggested to reduce exposure to air pollution (Dadvand et al. 2012b).

Our findings for the annual beach attendance were in line with those of green space playing time. Among the aforementioned potential underlying mechanisms for our observed associations between green space playing time and SDQ scores, physical activity and stress reduction could also be relevant to our findings for annual beach attendance.

Consistent with the inverse association between residential surrounding greenness and SDQ hyperactivity/inattention score, we estimated lower scores for ADHD and inattention symptoms in association with higher residential surrounding greenness. The etiology of ADHD is not well understood, but it is thought to be a multifactorial condition with both genetic and environmental factors playing roles in its pathogenesis. Maternal depression (Galéra et al. 2011; Sagiv et al. 2013) and exposure to air pollution (Chiu et al. 2013; Siddique et al. 2011) are among the reported nongenetic factors associated with ADHD. Therefore, the potential for green spaces to reduce stress and decrease exposure to air pollution might partly explain the associations between contact with green spaces and lower ADHD symptom scores. Moreover, these findings are in line with those of previous studies reporting improved attention in children who had moved to homes with higher surrounding greenness (Wells 2000) and “therapeutic effects” of playing in green spaces on ADHD symptoms (Kuo and Taylor 2004; Taylor and Kuo 2009; Taylor et al. 2001; van den Berg and van den Berg 2011).

Our findings for school greenness were consistent with those of residential surrounding greenness; however, none of the associations attained statistical significance. When we took both school and residential surrounding greenness (i.e., home–school greenness index) into account, estimated associations were similar to associations with residential surrounding greenness in terms of direction, strength, and statistical significance. When we repeated the analyses (fully adjusted models) by simultaneously including both residential surrounding greenness and school greenness in the models, we generally did not observe a notable change in associations for residential surrounding greenness in terms of direction and statistical significance; but for school greenness the associations were generally weaker than associations with school greenness alone (data not shown). These findings, together with the high correlation between residential surrounding greenness and the home–school greenness index, suggest that associations with the home–school greenness index may have been driven mostly by surrounding greenness at home, where children spend more time.

Residential proximity to a major green space was not associated with our investigated behavioral and ADHD indicators. This finding could be explained partly by our observed absence of association between residential proximity to a major green space and green space playing time, suggesting that in our study setting proximity to green spaces was not necessarily translatable to more use of green spaces. Our map of major green spaces did not address the quality of green spaces. Quality characteristics of green spaces such as aesthetics, biodiversity, walkability, sport/play facilities, safety, and organized social events have been suggested to predict the use of green spaces (McCormack et al. 2010) and could have an effect on our investigated associations between residential proximity to these spaces and our investigated outcomes.

Our study faced some limitations. We could not establish that the exposures preceded the outcomes because of the study’s cross-sectional design. We could not rule out the likelihood of self-selection bias, such that participants from higher SES groups may have been more likely to select living in greener neighborhoods and to have less likelihood of behavioral problems. Likewise, generalizability of our findings could have been affected by selection bias because BREATHE participants were different from nonparticipants with respect to a number of characteristics, including the prevalence of the outcomes. We used satellite-derived NDVI to assess surrounding greenness. Application of this objective measure of greenness enabled us to account for small-scale green spaces in a standardized way; however, NDVI does not distinguish between different types of vegetation, which could be relevant to our investigated associations. By using an NDVI map obtained at a single point in time (2011), we effectively assumed that the spatial distribution of NDVI across our study region remained constant over the study period (2012–2013). The findings of our previous study across the same region support the stability of the NDVI spatial contrast over seasons and years (Dadvand et al. 2012c). Moreover, data were not available for some potentially relevant confounders, such as parental mental health status. Furthermore, our behavioral questionnaires (i.e., SDQ and ADHD/DSM-IV) were rated by parents and teachers and were not validated by health professionals, which could have resulted in some degree of outcome misclassification. Although previous studies were generally supportive of an acceptable validity of SDQ (Becker et al. 2004; Goodman et al. 2000a; Mieloo et al. 2012), the reports of the validity of ADHD/DSM-IV questionnaires are not consistent (Willcutt et al. 2012). Besides, the Cronbach’s alpha for some of the SDQ subscales were slightly less than the 0.6 level for an acceptable internal consistency, so the results for these subscales need to be interpreted with caution.

## Conclusion

To date, the available body of epidemiological evidence evaluating potential impacts of green spaces on behavioral development in children is scarce. Our analyses showed that longer use of green spaces and higher residential surrounding greenness were associated with lower scores for SDQ total difficulties and most difficulties subscales. Furthermore, residential surrounding greenness was associated with reduced scores for ADHD and inattention symptoms. Moreover, more days spent at beaches was associated with lower scores for SDQ total difficulties and some difficulties subscales, and higher scores for the SDQ strength subscale. These findings support a beneficial impact of the use of green and blue spaces and residential surrounding greenness on behavioral development and ADHD in children. The findings for residential proximity to a major green space, a surrogate for access to green spaces, were not conclusive. Our study provides new insight regarding the potential health benefits of green and blue spaces, which can be of public health importance, considering the burden accompanying behavioral problems in children. Further longitudinal studies are required to confirm our findings and to address the quality of green spaces.

## Supplemental Material

(375 KB) PDF
