# Supplementary material for: Green and Blue Spaces and Behavioral Development in Barcelona Schoolchildren: The BREATHE Project
Source: Environ Health Perspect. 2014 Sep 9;122(12):1351–8. doi: 10.1289/ehp.1408215 (PMC4256702; doi:10.1289/ehp.1408215)
Supplement: (375 KB) PDF [file ehp.1408215.s001.508.pdf]

Environmental Health Perspectives doi:10.1289/ehp.1408215

Supplemental Material

Green and Blue Spaces and Behavioral Development in Barcelona Schoolchildren: The BREATHE Project

Elmira Amoly, Payam Dadvand, Joan Forns, Mònica López-Vicente, Xavier Basagaña, Jordi Julvez, Mar Alvarez-Pedrerol, Mark J Nieuwenhuijsen, and Jordi Sunyer

Green Spaces Questionnaire

The next questions are for obtaining information about the use of green spaces by your child. With green spaces, we mean areas like gardens and parks where there are vegetations and trees. Please do not consider the activity within the school promises during the school hours.

1- During the last **school period** (September-June) on average how many **times per week** and for each time how many **hours** did your child play in green spaces?

	School days	Weekends	Christmas, New Year, and Easter holidays
Time(s) per week			
Hour(s)			

2- During the last **summer holidays** (June-September) on average how many **times per week** and for each time how many **hours** did your child play in green spaces?

Time(s) per week	
Hour(s)	

Table S1. Median of NDVI averages across buffers of 100m, 250m, and 500m around home addresses separately for participants living within 300m of a major green space and those living further away.

NDVI	Residential Distance to major green spaces, ≤ 300m	Residential Distance to major green spaces, > 300m
100m buffer	0.084	0.040
250m buffer	0.110	0.046
500m buffer	0.113	0.049

Table S2. The medians of green space playing time, residential surrounding greenness (100m buffer), SDQ total difficulties score, and ADHD symptoms score in strata of parental educational achievements, employment, marital status, and ethnicity.

**Socioeconomic indicators**	**Median green space playing time**	**p-value[a]**	**Median residential surrounding greenness**	**p-value[a]**	**Median SDQ total difficulties score**	**p-value[a]**	**Median ADHD symptoms**	**p-value[a]**
**Maternal educational achievement**		< 0.01		0.77		< 0.01		< 0.01
No or primary education	336.0		0.044		10		8	
Secondary education	428.0		0.045		8		5	
University	518.5		0.044		7		3	
**Paternal educational achievement**		< 0.01		0.60		< 0.01		< 0.01
No or primary education	406.0		0.050		10		6	
Secondary education	425.0		0.043		8		5	
University	526.0		0.044		7		4	
**Maternal occupation**		< 0.01		0.43		< 0.01		0.02
Unemployed	429.0		0.042		9		6	
Employee	486.0		0.044		8		4	
Self-employed	517.0		0.047		7		4	
**Paternal occupation**		< 0.01		0.96		0.02		0.02
Unemployed	422.0		0.046		10		9	
Employee	462.0		0.043		7		4	
Self-employed	546.0		0.044		8		5	
**Ethnicity**		< 0.01		0.29		< 0.01		0.09
Non-Spanish	380.0		0.041		6		6	
Spanish	492.0		0.044		4		4	
**Parental marital status**		< 0.01		0.47		< 0.01		< 0.01
Single parent	404.0		0.042		9		7	
Non-single parent	496.0		0.045		8		4	

[a]*p*-value for Stata nptrend test.

Table S3. Adjusted[a] percent change (95% confidence interval) in outcomes associated with an IQR increase in green space playing time in models with and without the indicator of physical activity[b].

Outcome	Models without physical activity	Models with physical activity
**SDQ**		
Difficulties		
Total	-4.8 (-8.6, -0.9)**	-4.0 (-7.8, 0.0)**
Hyperactivity/inattention	-2.7 (-7.0, 1.5)	-2.0 (-6.4, 2.6)
Emotional symptoms	-8.2 (-13.9, -2.2)**	-7.0 (-12.9, -0.8)**
Conduct problems	0.7 (-5.6, 7.5)	0.0 (-6.4, 6.9)
Peer relationship problems	-15.4 (-22.7, -7.4)**	-12.8 (-20.4, -4.4)**
Strengths		
Prosocial behavior	0.2 (-1.0, 1.5)	-0.1 (-1.4, 1.2)
**ADHD/DSM-IV**		
ADHD	-1.6 (-9.0, 6.4)	-0.6 (-8.1, 7.5)
Inattention	-0.2 (-7.7, 7.9)	1.7 (-6.1, 10.0)
Hyperactivity	-5.4 (-14.5, 4.8)	-5.4 (-14.7, 4.9)

[a]Adjusted for child's school level, sex, ethnicity, preterm birth, breastfeeding, exposure to environmental tobacco smoke, maternal smoking during pregnancy, responding person, parental educational achievement, parental employment status, parental marital status and neighborhood socioeconomic. [b]The frequency of physical exercise that could make the child sweating or breathless (categories: once or less/2-3 times/4-7 time per week).

Table S4. Adjusted[a] percent change (95% confidence interval) in outcomes associated with living within 300m of any green space regardless of its size, Barcelona, 2011.

**Outcome**	**% change (95% CI)**
**SDQ**	
Difficulties	
Total	-4.7 (-14.8, 6.6)
Hyperactivity/inattention	6.4 (-6.7, 21.4)
Emotional symptoms	-8.7 (-23.3, 8.6)
Conduct problems	-12.3 (-26.5, 4.5)
Peer relationship problems	-15.3 (-33.2, 7.3)
Strengths	
Prosocial behavior	-1.5 (-5.0, 2.1)
**ADHD/DSM-IV**	
ADHD	-10.1 (-26.9, 10.7)
Inattention	-13.4 (-29.5, 6.4)
Hyperactivity	-3.4 (-26.8, 27.6)

[a]Adjusted for child's school level, sex, ethnicity, preterm birth, breastfeeding, exposure to environmental tobacco smoke, maternal smoking during pregnancy, responding person, parental educational achievement, parental employment status, parental marital status and neighborhood socioeconomic.
